# Two Novel Variants in the *ATRX* Gene Associated with Variable Phenotypes

**DOI:** 10.1155/2019/2687595

**Published:** 2019-11-06

**Authors:** D. Hettiarachchi, B. A. P. S. Pathirana, P. J. Kumarasiri, V. H. W. Dissanayake

**Affiliations:** Human Genetics Unit, Faculty of Medicine, University of Colombo, Sri Lanka

## Abstract

The X-linked alpha-thalassemia mental retardation (ATR-X) syndrome is a rare genetic condition caused by mutations in the X‐encoded gene *ATRX*. Here we describe two unrelated patients of Sri Lankan origin with novel missense variants in the *ATRX* gene: c.839C>T|p.Cys280Tyr and c.5369C>T|p.Ala1790Val. These two novel variants were associated with variable phenotypes which clinically resembled X-linked mental retardation-hypotonic facies syndrome and Smith-Fineman-Myers syndrome respectively. These cases expand the clinical spectrum of ATR-X syndrome and open new opportunities for the molecular diagnosis of *ATRX* mutations in male patients with severe global developmental delay and intellectual disabilities.

## 1. Introduction

The X-linked alpha-thalassemia mental retardation (ATR-X) syndrome is a rare genetic condition caused by mutations in the X‐encoded gene *ATRX*. It is known to show a wide spectrum of clinical manifestations such as alpha thalassemia, developmental delay, genital abnormalities, gastrointestinal disorders and epilepsy in variable degrees [1, 2]. The *ATRX *gene spans about 300 kb genomic DNA and is composed of 35 exons [3, 4]. It encodes for the ATRX nuclear protein (2,492 residue; 280 kDa) which is predominantly localized to heterochromatin and nuclear PML bodies [2, 5]. This is a chromatin-associated protein, a member of the SNF2 (SWI/SNF) family of chromatin remodeling factors. It contains two main highly conserved domains. Missense mutations that give rise to ATR-X syndrome fall mainly within these two domains [6]. At the N-terminus is the ATRX-DNMT3-DNMT3L (ADD) domain, which is a plant homeodomain (PHD)-like zinc finger with an additional C2-C2 motif. At the C terminus is a helicase/ATPase domain. The structure of the ADD domain has recently been solved and shows that it is highly related to the zinc finger domain of DNMT3 family of de novo DNA methyltransferases. ATRX acts as a DNA-dependent ATPase and as a DNA translocase, it confers modest chromatin-remodeling activity in vitro [7, 8]. Here we describe two novel missense variants in the ATRX gene with variable phenotypes.

## 2. Case Presentation

Here we describe two unrelated patients with novel missense variants in the *ATRX* gene. c.839C>T|p.Cys280Tyr and c.5369C>T|p.Ala1790Val. The first patient is a 7 years old male born to nonconsanguineous parents. He had another male sibling who died at the age of 9 months and had the following comorbidities: Global developmental delay and primary immune deficiency syndrome. He succumbed to death due to pneumonia at 9 months. The proband also has global developmental delay, microcephaly, mental retardation, brachydactyly, scoliosis, a broad nasal bridge, hoarse voice, carp-like mouth, low set ears, and hyperactivity. His investigations including CT brain, full blood count and karyotype (46, XY) were normal. 2D echo showed a situs solitus mesocardia which was normal on follow up.

Whole exome sequencing was performed as follows; Peripheral blood samples of the proband and the family members were collected to EDTA tubes following written informed consent. Proband's genomic DNA was extracted from the blood leucocytes using QIAamp DNA Mini Kit according the manufacturer's protocol (https://www.qiagen.com/ch/resources/download.aspx?id=62a200d6-faf4-469b-b50f-2b59cf738962&lang=en). Extracted DNA was then subjected to whole exome sequencing on an Illumina HiSeq platform followed by library preparation using the Agilent SureSelect Human All Exon + UTR kit according to the manufacturer's protocol (https://www.agilent.com/cs/library/datasheets/public/SureSelect%20V6%20DataSheet%205991-5572EN.pdf).

Genetic analysis of the paired end sequencing data was performed using an in-house bioinformatics pipeline. Obtained FASTQ files were mapped with the GrCh37 human reference sequence using BWA‐mem algorithm and Genome Analysis Tool Kit (GATK). The annotation of the VCF file was performed using SNP-eff with Refseq, clinical databases and population frequency databases. NCBI's “common and no known medical impacts” database (ftp://ftp.ncbi.nlm.nih.gov/pub/clinvar/vcf_GRCh37/), Genome Aggregation Database (gnomAD, http://gnomad.broadinstitute.org/) and the Exome Aggregation Consortium (ftp://ftp.broadinstitute.org/pub/ExAC_release/release0.2/) were used.

**Figure 1:**
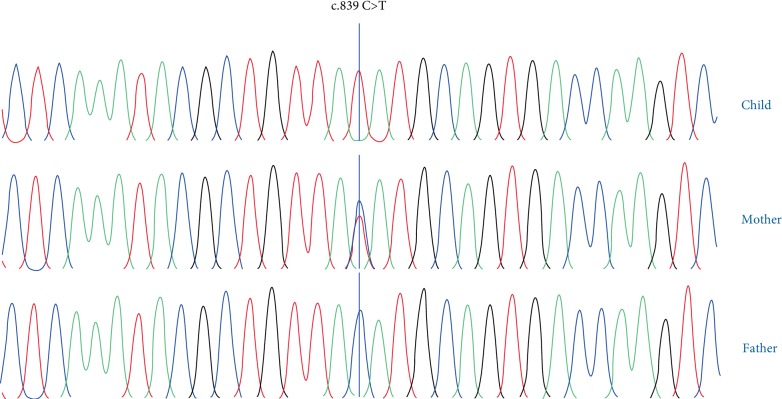
Sanger sequencing results of the child (hemizygous for the variant), mother (heterozygous for the variant) and father (hemizygous for the ancestral allele) respectively.

All reportable sequence variants were confirmed by visual inspection of the alignment. The following tools were used for in-silico functional prediction (Mutation Taster: Disease Mutation; Polyphen2 SIFT). The variant c.839C>T was predicted as damaging and was classified as likely to be pathogenic. Sanger sequencing was done to confirm the presence of the variants in family members. It was confirmed that the child was hemizygous and the mother was heterozygous for the variant (Figure 1). Clinically he was diagnosed as having X-linked mental retardation-hypotonic facies syndrome.

The second patient is a 12-year-old male born to nonconsanguineous parents. He has a healthy younger 3-year-old sister. The proband had the following clinical features; severe developmental delay, learning difficulties and generalized tonic clonic seizures. Currently he is on sodium valproate for seizures. However, the control is not satisfactory. He has self-injurious and sensory seeking behavioral abnormalities. MRI brain showed minimal degrees of cerebral and cerebellar atrophy. He also has motor abnormities such as dystonia.

Trio exome sequencing was performed on the proband and the parents following the same protocol as above. The missense variant ATRX: c.5369C>T, p. Ala1790Val was found in the heterozygous state in the mother and in hemizygous state in the proband. The results were confirmed using bi-directional genomic sequencing using a familial positive control for the sequence variant. Methylation analysis was also performed using a previously described protocol [9]. The proband showed completed X inactivation and the mother of the proband highly skewed X-inactivation. The amplification results from digested and nondigested DNA from the patient's mother showed highly skewed (nonrandom) X-inactivation. Which is a common finding among X-linked mental retardation female carriers confirmed using the human androgen receptor (AR) gene located at Xq11.2. The human AR gene contains a highly polymorphic in-frame CAG repeat encoding 11–31 glycine residues in exon 1 of the gene [10].

No other clinically relevant mutations were found in both patients and in any of the family members who were sequenced. Consent to publish was taken from the patients and family members following a protocol approved by the Ethics Review Committee, Faculty of Medicine University of Colombo.

## 3. Discussion

**Figure 2:**
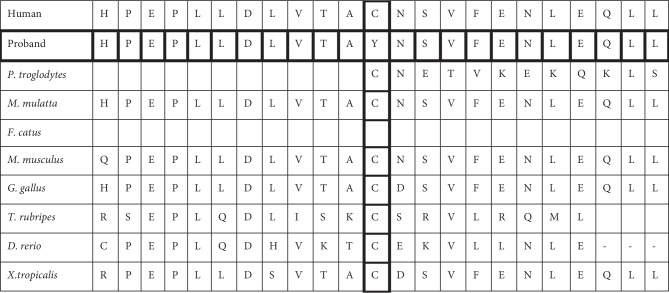
Evolutionary conservation of the mutated residue (p.Cys 280Tyr).

Thus far, among the variants reported in the *ATRX* gene associated with X-linked mental retardation-hypotonic facies syndrome (https://www.omim.org/entry/300032#41) the two variants described here are not reported elsewhere. The first variant c.839C>T|p.Cys280Tyr is located in exon 9 of the *ATRX* gene. This variant causes a nonconservative substitution of amino acids, i.e., substitution of Cysteine by Tyrosine that may result in a significant alteration of the structure of the protein. As it lies in the ADD domain this nonconservative amino acid substitution is likely to be pathogenic [11]. ATRX mutations cluster mainly in the ADD (50%) and helicase (30%) domains [7] and these correlate with the regions of highest sequence conservation between human and mouse (Figure 2). It has been reported that mutations in the ADD domain correlate with severe psychomotor impairment and severe urogenital abnormalities. However, our patient had mental retardation but was not reported to have any urogenital abnormalities. A phenotype comparison was made (Table 1) with our patients and the commonly associated clinical features of ATRX-syndrome [13].

**Figure 3:**
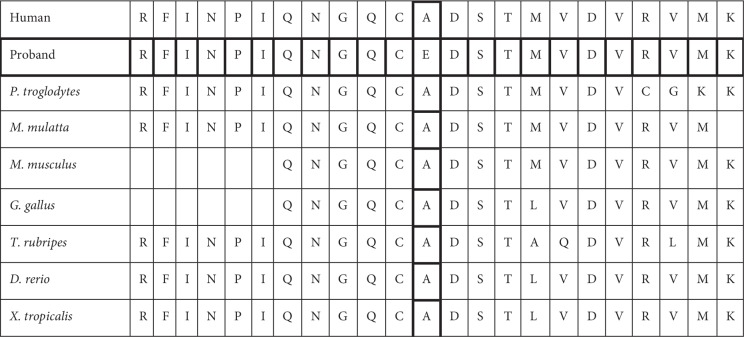
Evolutionary conservation of the mutated residue (p. Ala1790Val).

The second variant c.5369C>T|p.Ala1790Val resides outside the canonical dinucleotide splice donor and acceptor sites for ATRX. Independent splice site prediction algorithms failed to identify any significant change to the predicted normal splicing behaviour that might indicate a cryptic splice site. This theoretical analysis should be supported by further functional work to ascertain its exact pathogenicity. Furthermore, the ATRX gene forms a complex with DAXX which is a histone H3.3 chaperone [14]. It was found that ATRX–DAXX complex participates in chromatin remodeling for genes that are controlled by DAXX-interacting sequence-specific transcription factors [2]. Given that the *ATRX* gene is highly conserved across species (Figures 2 and 3) the resultant mutant residue may cause inappropriate regulation of these target genes.

The complexity of the disease spectrum suggests that ATRX could be involved in the regulation of other as yet unidentified genes [3]. The first patient had 5 of the commonly associated clinical features while the second proband had only 2 out of the 12 main clinical features of ATR-X syndrome. Both these patients were negative for alpha thalassemia and HbH inclusions which were seen in 87% of the patients with ATR-X [13]. In silico, functional prediction tools identified the c.839C>T variant to be damaging however c.5369C>T was shown to have an uncertain clinical significance. There has been previous reports of mental retardation and epilepsy co-existing in patients with variants in *ATRX *gene [14]. These cases expand the clinical spectrum of ATR-X syndrome and open new opportunities for the molecular diagnosis of *ATRX* mutations in male patients with severe global developmental delay and intellectual disabilities.

## Figures and Tables

**Table 1:** Comparison of clinical findings in ATR-X syndrome [12] and the probands.

Clinical feature	Incidence reported in ATR-X syndrome	Proband 1	Proband 2
Profound mental retardation	95%	Yes	Yes
Characteristic facial features	94%	Yes	Very subtle dysmorphisms
Skeletal abnormalities	91%	Yes, Brachydactyly and scoliosis	
HbH inclusions	87%	No	No
Motor abnormalities such a neonatal hypotonia	85%	No	Dystonia
Genital abnormalities	80%	No	No
Microcephaly	76%	Yes	No
Gut dysmotility	75%	No	No
Short stature	66%	No	No
Seizures	35%	No	Yes - GTC
Cardiac defects	18%	Situs solitus mesocardia which was normal on follow up	No
Renal and urinary abnormalities	14%	No	No
Other findings	—	Hoarse voice, behavioural problems	MRI-minimal degrees of cerebral and cerebellar atrophy, behavioural problems
